# Synthesis, Structural, and Adsorption Properties and Thermal Stability of Nanohydroxyapatite/Polysaccharide Composites

**DOI:** 10.1186/s11671-017-1911-5

**Published:** 2017-02-27

**Authors:** Ewa Skwarek, Olena Goncharuk, Dariusz Sternik, Wladyslaw Janusz, Karolina Gdula, Vladimir M. Gun’ko

**Affiliations:** 1 Department of Radiochemistry and Colloids Chemistry, Faculty of Chemistry, Maria Curie-Sklodowska University, M. Curie-Sklodowska Sq. 3, 20-031 Lublin, Poland; 2 Chuiko Institute of Surface Chemistry, National Academy of Science of Ukraine, 17 General Naumov Street, 03164 Kiev, Ukraine; 3 Department of Physicochemistry of Solid Surface, Faculty of Chemistry, Maria Curie-Sklodowska University, M. Curie-Sklodowska Sq. 3, 20-031 Lublin, Poland; 4 Department of Theoretical Chemistry, Faculty of Chemistry, Maria Curie-Sklodowska University, M. Curie-Sklodowska Sq. 3, 20-031 Lublin, Poland

**Keywords:** Nanohydroxyapatite, High- and low-esterified pectins, Agar, Sodium alginate, Chitosan, Composites, Sr(II) adsorption

## Abstract

A series of composites based on nanohydroxyapatite (nHAp) and natural polysaccharides (PS) (nHAp/agar, nHAp/chitosan, nHAp/pectin FB300, nHAp/pectin APA103, nHAp/sodium alginate) was synthesized by liquid-phase two-step method and characterized using nitrogen adsorption–desorption, DSC, TG, FTIR spectroscopy, and SEM. The analysis of nitrogen adsorption–desorption data shows that composites with a nHAp: PS ratio of 4:1 exhibit a sufficiently high specific surface area from 49 to 82 m^2^/g. The incremental pore size distributions indicate mainly mesoporosity. The composites with the component ratio 1:1 preferably form a film-like structure, and the value of *S*_BET_ varies from 0.3 to 43 m^2^/g depending on the nature of a polysaccharide. Adsorption of Sr(II) on the composites from the aqueous solutions has been studied. The thermal properties of polysaccharides alone and in nHAp/PS show the influence of nHAp, since there is a shift of characteristic DSC and DTG peaks. FTIR spectroscopy data confirm the presence of functional groups typical for nHAp as well as polysaccharides in composites. Structure and morphological characteristics of the composites are strongly dependent on the ratio of components, since nHAp/PS at 4:1 have relatively large *S*_BET_ values and a good ability to adsorb metal ions. The comparison of the adsorption capacity with respect to Sr(II) of nHAp, polysaccharides, and composites shows that it of the latter is higher than that of nHAp (per 1 m^2^ of surface).

**Electronic supplementary material:**

The online version of this article (doi:10.1186/s11671-017-1911-5) contains supplementary material, which is available to authorized users.

## Background

In recent years, intense researches are carried out to prepare bio-hydroxyapatite composites with desired biological, physical, and mechanical properties. Hydroxyapatite and its composites are of interest due to applications in medicine. The physicochemical properties and biocompatibility make them a very attractive object for investigations both in vivo and in vitro [1–19].

Modification of hydroxyapatite (HAp) with such natural polysaccharides as chitosan [3–6], sodium alginate [7–13], agar [14, 15] and pectins [16–19], or embedding HAp nanoparticles into a polymer matrix as a filler allows one to control the morphological, structural, and mechanical properties of composites to enhance the functional use. The composites based on chitosan with HAp or alginate are mainly used to treat bone implants [3–8, 11–14, 16–18] or to use as drugs carriers [9, 10, 15].

Analysis of the literature shows that the synthesis of HAp/PS composites, their use, and control of the properties are far from exhausted ones. The use of HAp/PS composites as adsorbents could be promising since the components alone show a high adsorption capacity with respect to heavy metal cations [20–34]. Creation of composites allows one to control the structure of the materials to improve the morphology and to enhance the adsorption properties. It is known that natural polysaccharides are good sorbents of some kinds of dyes [29–31, 35] and heavy metal ions [23–33] because of specific interactions of the amino and hydroxyl groups with adsorbates [37]. The amino groups of polysaccharides can be cationized that allows the effective adsorption of ionic dyes [38]. However, the use of some polysaccharides in their native form is difficult since the viscosity of the solutions is high even at a low concentration because of tendency to gelling. Therefore, composites with PS can be more appropriate for the adsorption applications due to diminution of the mentioned negative effects [39–41]. Immobilization of macromolecules on a HAp surface allows an increase in sorption activity of the composites compared to the components alone.

The objective of this work was the synthesis of nHAp/polysaccharide composites and the study of the structural and morphological characteristics, the thermal behavior, and sorption capacity with respect to Sr(II).

## Methods

### Materials

The nHAp/PS composites were synthesized by mixing of nHAp suspensions with polysaccharides solutions in two stages. The first stage was the synthesis of nHAp by a wet chemical method. In the reaction

1 $$ 10\ \mathrm{C}\mathrm{a}{\left(\mathrm{OH}\right)}_2 + 6\ {\mathrm{H}}_3{\mathrm{PO}}_4\kern0.5em \to\ {\mathrm{Ca}}_{10}{\left({\mathrm{PO}}_4\right)}_6{\left(\mathrm{OH}\right)}_2 + 18\ {\mathrm{H}}_2\mathrm{O} $$

calcium hydroxide (Aldrich) and phosphoric acid (POCh, Gliwice) were used as 1 M aqueous solutions of 0.18 and 0.3 L, respectively. The H_3_PO_4_ solution was dropped into the Ca(OH)_2_ suspension placed in a flask for 15 min. While dropping the reaction, mixture was stirred vigorously and then dried in a dryer at 80 °C for 24 h. A white sediment with crystalline hydroxyapatite was obtained. Then the sediment was washed with redistilled water till the constant value of redistilled water conductivity was achieved. The average crystallite size determined from XRD patterns using Scherrer’s equation applied to a peak at 2θ = 25.9 was 26 nm. The degree of crystallinity [42] was 22%.

The second stage was the synthesis of nHAp/polysaccharide composites using chitosan (deacetylation degree 82%, “Bioprogress” CJSC, Moscow, Russia), high-etherified apple pectin APA 103 at the degree of etherification (DE) of 66–68% and low-etherified apple pectin APA 300 FB with galacturonic acid with free carboxyl groups 64–69% (Andre Pectin, China), and sodium alginate (SA) at a mass fraction of the basic substance of 99.0% (China) as received. nHAp composites were prepared by mixing of the nHAp suspension and PS solution. Additionally, the polysaccharide solution (2 wt.%) and nHAp suspension (4 wt.%) sonicated for 3 min were prepared using distilled water, mixed at the nHAp/PS ratio of 1:1 and 4:1, and stirred for 30 min. Then the nHAp/PS suspensions were dried at 40 °C in air.

The hydroxyl groups are the main functional groups of PS, which can be esterified or oxidized. The carboxyl groups of uronic acid can be esterified, and the amino groups of amino sugars can be acylated. Modified PS are capable to create strong complexes with metal ions, as well as with polar low-molecular weight organics.

The formation of composites occurs due to strong interactions of the phosphate and hydroxyl groups of nHAp with the COO^−^, OH, and other polar groups in PS [18]. The polysaccharide molecules also tend to form the hydrogen bonds with each other resulting in gelation of their aqueous solutions upon heating at certain temperatures. The calcium phosphate ions can be trapped in the PS chains. The cross-linking reactions may occur in the composites. Therefore, nHAp nanoparticles could be well distributed in the PS network and remained in stable state for a long period.

### Fourier Transform Infrared (FTIR) Spectroscopy

FTIR spectra of powdered samples (grinded with dry KBr at the mass ratio 1:9) over the 4000–400 cm^−1^ range were recorded using a ThermoNicolet FTIR spectrometer with a diffuse reflectance mode.

### Scanning electron microscopy (SEM)

The surface morphology of composites was analyzed using field emission scanning electron microscopy employing a QuantaTM 3D FEG (FEI, USA) apparatus operating at the voltage of 30 kV.

### Textural characteristics

Specific surface areas and pore volumes were determined from low-temperature nitrogen adsorption isotherms using a Micromeritics ASAP 2020 or 2405N adsorption analyzer. Before measurements, the samples were outgassed at 80 °C for 12 h. The nitrogen desorption data were used to compute the pore size distributions (PSD, differential *f*_V_ ~ d*V*_p_/d*R* and *f*_S_ ~ d*S*/d*R*) using a self-consistent regularization (SCR) procedure under non-negativity condition (*f*_V_ ≥ 0 at any pore radius *R*) at a fixed regularization parameter *α* = 0.01 using a model of voids (V) between spherical nonporous nanoparticles packed in random aggregates (V/SCR model) [43]. The differential PSD with respect to the pore volume *f*_V_ ~ d*V*/d*R*, ∫ *f*_V_d*R* ~ *V*_p_ were re-calculated to incremental PSD (IPSD) at Φ_V_(*R*_*i*_) = (*f*_V_(*R*_*i*+1_) + *f*_V_(*R*_*i*_))(*R*_*i*+1_ − *R*_*i*_)/2 at ∑Φ_V_(*R*_*i*_) = *V*_p_. The *f*_V_ and *f*_S_ functions were also used to calculate contributions of micropores (*V*_micro_ and *S*_micro_ at 0.35 nm < *R* < 1 nm), mesopores (*V*_meso_ and *S*_meso_ at 1 nm < *R* < 25 nm), and macropores (*V*_macro_ and *S*_macro_ at 25 nm < *R* < 100 nm).

### Thermal analysis

Thermal analysis was carried out using a STA 449 Jupiter F1 (Netzsch, Germany) apparatus, sample mass ~16 mg placed into a corundum crucible, air flow of 50 mL min^−1^, a heating rate of 10 °C min^−1^, temperature range of 30–950 °C, and S TG-DSC sensor thermocouple type. Empty corundum crucible was used as a reference. The gaseous products emitted during decomposition of the materials were analyzed by using a FTIR Brucker (Germany) spectrometer and QMS 403D Aëolos (Germany) coupling on-line to STA instrument. The QMS data were gathered in the range from 10 to 200 a.m.u. The FTIR spectra were recorded in the range of 4000–600 cm^−1^ with 16 scans per spectrum at a resolution of 4 cm^−1^.

### Adsorption of Sr(II)

The adsorption of Sr(II) ions vs. pH at the composite/electrolyte solution interface was determined by the means of the radioisotope method. The initial concentration of Sr(II) ions was 10^−4^ M. NaCl (0.001 mol/dm^3^) was used as a background electrolyte, and pH was changed from 3 to 10. The adsorption measurements were complemented by the potentiometric titration of the composites in the suspensions and the electrophoresis measurements. The adsorption measurements were performed in a thermostated Teflon vessel at 25 °C. To eliminate CO_2_, all the potentiometric measurements and adsorption experiments were carried out under the nitrogen atmosphere. The pH values were measured using a set of glass REF 451 and calomel pHG201-8 electrodes with Radiometer assembly. Radioactivity of the solutions before and after adsorption was measured using a LS 5000 TD Beckmann liquid scintillation counter. Because ^90^Sr decays to the radioactive ^90^Y, the measurements were carried out in two channels in order to calculate radioactivity of ^90^Sr.

## Results and Discussion

### Textural Characterization

**Fig. 1:**
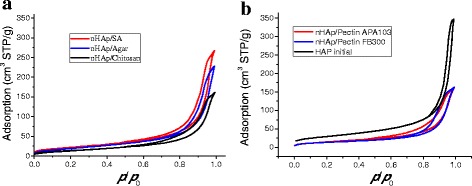
Nitrogen adsorption–desorption isotherms for composites with nHAp:PS ratio 4:1

The BET surface area and pore volume of composites (Table 1) depend on the content and type of PS. The initial nHAp has S_BET_ of 105 m^2^/g and *V*_p_ of 0.54 cm^3^/g, while for composites, they decrease with increasing PS concentration due to filling of inter-particle voids in aggregates by polymer molecules. The shape of the nitrogen adsorption–desorption isotherms (Fig. 1) corresponds to type II with hysteresis loop H3 of the IUPAC classification [44, 45] corresponding to the textural porosity of aggregates of nonporous nanoparticles.

**Table 1:** Textural characteristics of initial nHAp and nHAp/PS nanocomposites

Sample	*S*_BET_ (m^2^/g)	*S*_micro_ (m^2^/g)	*S*_meso_ (m^2^/g)	*S*_macro_ (m^2^/g)	*V*_p_ (cm^3^/g)	*V*_micro_ (cm^3^/g)	*V*_meso_ (cm^3^/g)	*V*_macro_ (cm^3^/g)	*R*_p,V_ (nm)
nHAp	106	7.3	85	14	0.54	0.004	0.35	0.19	22.3
nHAp/agar 4:1	75	1.2	71	2.9	0.35	0.001	0.31	0.05	17.0
nHAp/agar 1:1	43	14	29	0.1	0.26	0.009	0.24	0.05	11.3
nHAp/SA 4:1	82	6.4	73	2.6	0.42	0.002	0.36	0.05	16.7
nHAp/SA 1:1	1	0.0	0.8	0.2	0.10	0.0	0.08	0.02	63.6
nHAp/chitosan 4:1	53	0.1	52	1.3	0.25	0.0	0.23	0.02	15.4
nHAp/chitosan1:1	8	0.3	6.2	1.2	0.11	0.0	0.03	0.08	63.4
nHAp/FB300 4:1	49	7.4	41	0.9	0.25	0.003	0.23	0.02	14.7
nHAp/FB300 1:1	0.3	0.1	0.0	0.2	0.00	0.001	0.00	0.001	88.6
nHAp/APA103 4:1	56	0.4	55	1.1	0.25	0.0	0.23	0.02	13.9

Specific surface area in total (*S*_BET_), micropores (*S*_micro_), mesopores (*S*_meso_), and macropores (*S*_macro_) and respective pore volumes (*V*_p_, *V*_micro_, *V*_meso_, *V*_macro_). *R*_p,V_ is the average pore radius with respect to the pore volume

The hysteresis loop shape indicates dominant contribution of mesopores (filled by adsorbed nitrogen during the measurements). It should be noted that in the case of highly disperse materials, only a certain part of pores can be filled by nitrogen because large macropores remain empty, i.e., *V*_p_ < *V*_em_ = 1/*ρ*_b_ − 1/*ρ*_0_, where *ρ*_b_ and *ρ*_0_ are bulk and true densities of the materials.

**Fig. 2:**
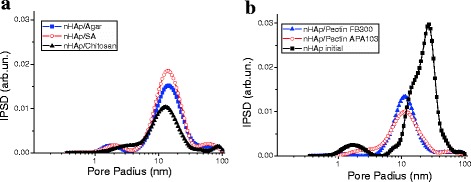
Incremental pore size distributions for composites with ratio nHAp:PS 4:1

**Fig. 3:**
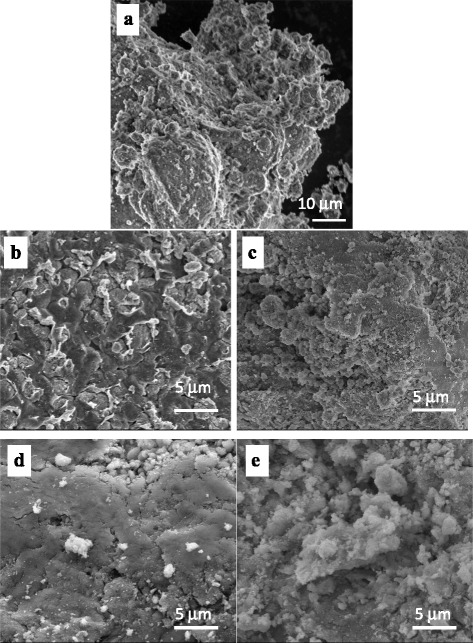
SEM images of initial nHAp (**a**), nHAp/chitosan (**b**) 1:1 and (**c**) 1:4 and nHAp/agar (**d**) 1:1 and (**e**) 1:4

**Fig. 4:**
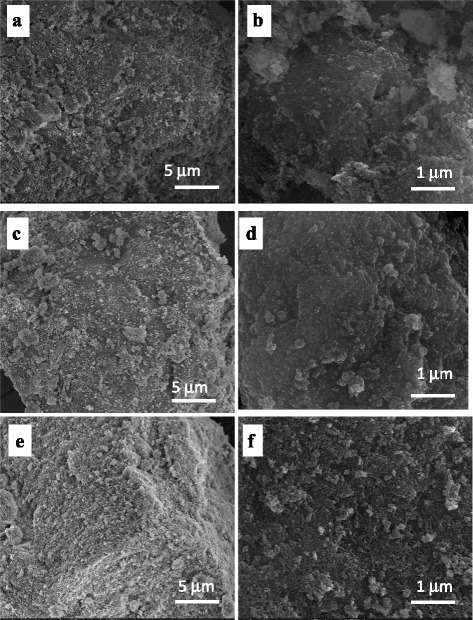
SEM images of nHAp/pectin FB 300 (**a**, **b**), nHAp/pectin APA103 (**c**, **d**), nHAp/SA (**e**, **f**) components ratio 1:4

The pore size distribution functions (Fig. 2) confirm the conclusion based on the isotherm shape (Fig. 1) that the composites are mainly mesoporous, since contributions of micropores and macropores are small (Table 1). The first peak of the PSD corresponds to narrow voids between nanoparticles/polymers closely located in the same aggregates. Broader voids can be between neighboring aggregates. The PSD show that different PS form different shells of nanoparticles, especially in the range of narrow pores at *R* < 10 nm (Fig. 2). Therefore, the average pore radius *R*_p,V_ in nHAp/PS at the ratio of 4:1 is not the same, and it is in the range of 13.9–17.0 nm corresponding to mesopores (Table 1). Despite filling of voids by PS, the values of *R*_p,V_ increase with increasing PS content. These changes can be explained by several factors. First, narrow voids are more strongly filled by PS than broad voids. Second, adsorption of PS results in compacting of aggregates of nanoparticles and agglomerates of aggregates (see Figs. 1, 2, 3, and 4, Table 1).

A film-like, near-monolithic structure is formed in nHAp/PS at the ratio of 1:1 (Fig. 3b, d) as evidenced by low values of the specific surface area. However, some porosity of the composites remains. At the component ratio of 4:1, the structure of composites is more porous and can be described as multimodal aggregates of nHAp/PS with sizes over a wide range of 20–250 nm (Fig. 3c, e). Similar structure of aggregates of primary particles inherent to initial nHAp (Fig. 3a), and it remains for composites nHAp/pectins and nHAp/SA (Fig. 4). Thus, a relatively high value of *S*_BET_ and porous structure of these composites (Table 1, Figs. 1, 2, 3, and 4) indicate the prospects for their use as better adsorbents than those at 50 wt.% of PS.

### Fourier Transform Infrared Spectroscopy (FTIR)

**Fig. 5:**
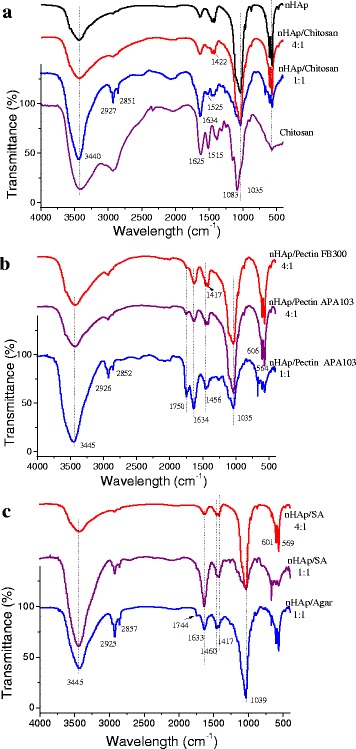
FTIR spectra of samples of nHAp/PS composites: (**a**) nHAp/chitosan, (**b**) nHAp/Pectin, and (**c**) nHAp/SA and nHAp/Agar

The IR spectrum of hydroxyapatite (Fig. 5) exhibits characteristic bands at 561 and 602 cm^−1^ corresponding to triply degenerated bending modes of the O–P–O bond vibrations in the phosphate groups [46–49]. A band at 472 cm^−1^ corresponds to double-degenerated bending modes of the O–P–O bonds [46–48]. A band at 962 cm^−1^ can be attributed to non-degenerated symmetric stretching modes of the P–O bonds [46–50]. Bands at 1032 and 1101 cm^−1^ are due to triply degenerated asymmetric stretching vibrations of the P–O bonds.

The presence of (CO_3_)_2_ groups is confirmed by bands at 1414 cm^−1^, which are assigned to stretching mode of the (CO_3_)_2_ groups [46, 51–53], and at 1465 cm^−1^, which can be attributed to the stretching modes of the (CO_3_)_2_ groups in A-type of carbonated apatite [46, 54, 55].

OH groups and adsorbed water molecules, presented in the hydroxyapatite sample, give peaks at 875 cm^−1^ (hydrogen phosphate groups) and 2856–3656 cm^−1^ with a maximum at 3400 cm^−1^.

All characteristic bands of hydroxyapatite remain in the IR spectra of the nHAp/PS composites (Fig. 5), but their intensity decreases with decreasing content of hydroxyapatite. The appearance the bands at 2927 and 2851 cm^−1^ is due to C–H asymmetric and symmetric stretching vibrations in the aliphatic CH_2_ groups of PS. Especially noticeable increase in intensity of broad band with a maximum at 3445 cm^−1^ is due to the OH groups in PS forming the hydrogen bonds with each other or adsorbed water molecules. Additionally, the N–H bonds in the amino groups of chitosan give the bands at ~3350 cm^−1^. Similar features of the IR spectra of nHAp/pectin are shown in Fig. 5b. Pectin molecules include few hundred linked galacturonic acid residues forming a long molecular chain with polygalacturonic acid, wherein a fraction of galacturonic acid subunits is methoxylated. The pectin molecules contain a large amount of carboxyl (free and esterified), hydroxyl, methoxyl, and acetyl groups. The bands at 2926 and 2852 cm^−1^ of the C–H stretching vibrations and a broad band with a maximum at 3445 cm^−1^ increase with increasing content of pectins. The IR spectra of pectins are characterized by bands at 1750–1700 cm^−1^ related to the stretching vibrations of the carbonyl, ester, and carboxyl groups. The IR spectra of nHAp/sodium alginate (Fig. 5c) show similar bands of the hydroxyl, ether, and carboxylic groups, as well as the O–H and C–H stretching vibrations of alginate. Bands at 1633 and 1460 cm^−1^ can be attributed to the asymmetric and symmetric stretching vibrations of carboxylate salt ions. These bands can be used to characterize structures of alginate, its derivatives, and ingredients.

### Thermal Analysis

**Fig. 6:**
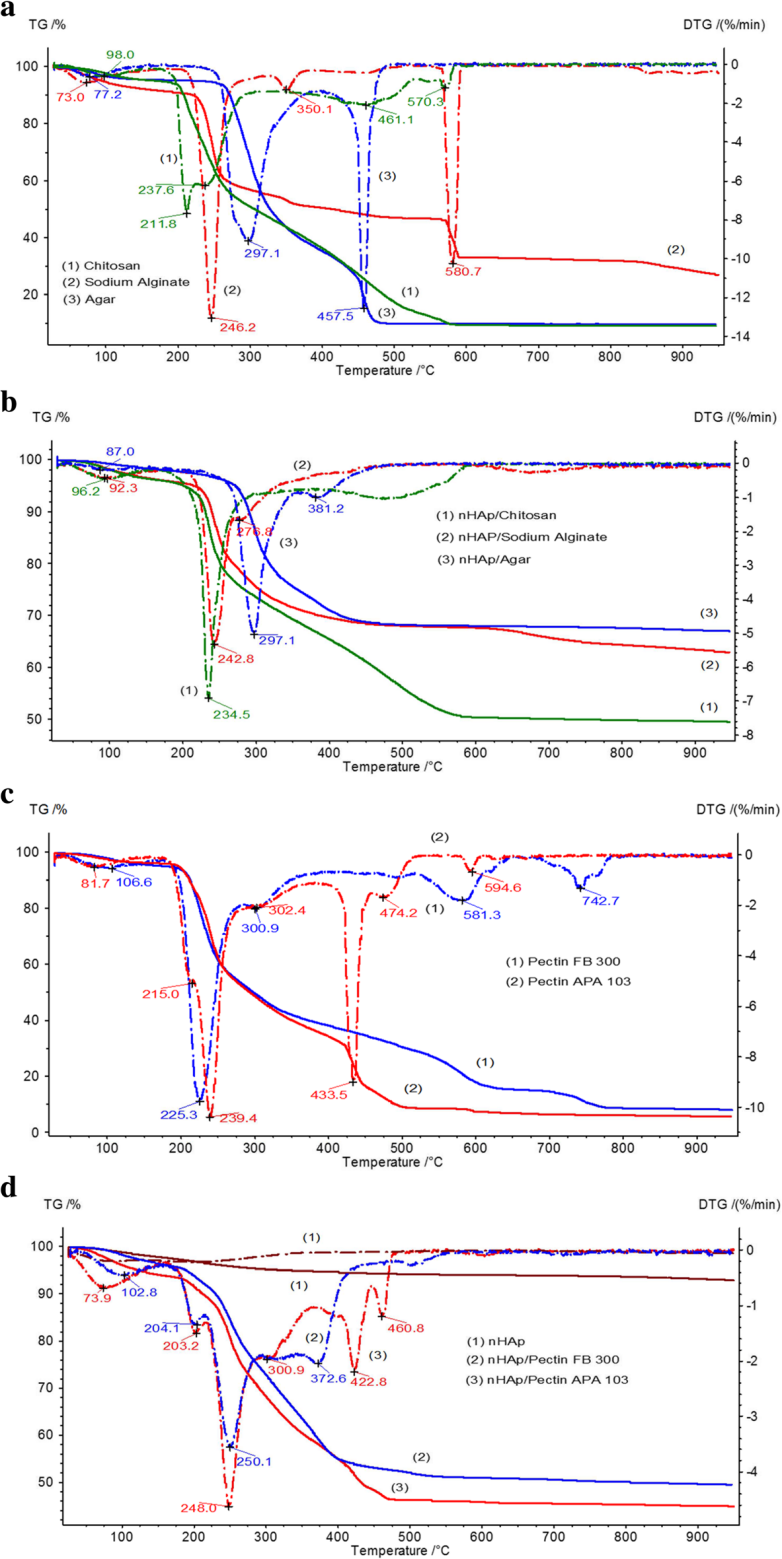
DTG (*broken curve*) and TG (*continuous curve*) for initial PS and nHAp/PS composites. **a** Initial chitosan, sodium alginate, and agar. **b** nHAp/Chitosan, nHAp/SA, and nHAp/Agar composites. **c** Pectin FB300 and pectin APA103. **d** nHAp/pectin FB300 and nHAp/pectin APA103 composites

**Fig. 7:**
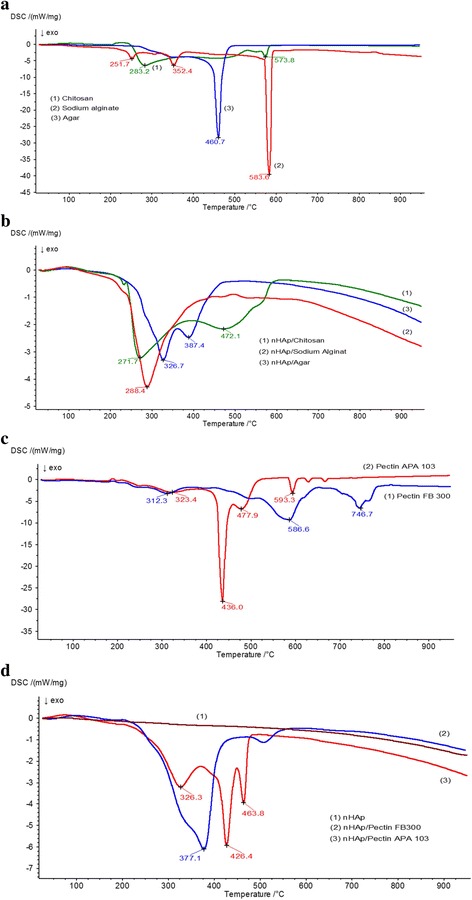
DSC curves for initial PS and nHAp/PS composites. **a** Initial chitosan, sodium alginate, and agar. **b** nHAp/chitosan, nHAp/SA, and nHAp/agar composites. **c** Pectin FB300 and pectin APA103. **d** nHAp/pectin FB300 and nHAp/pectin APA103 composites

The thermal characteristics (TG, DTG, and DSC) of nHAp/PS, nHAp, and polysaccharides were studied upon heating of samples in air (Figs. 6 and 7, Additional file 1: Table S1). Our previous studies [56] have shown that in case of thermal decomposition of hydroxyapatite (Fig. 6d), the weight losses are results of the process of desorption of physically adsorbed water and dehydroxylation in temperature range to 200 °C and removing of carbonates at higher temperatures. According to literature [57–59], the thermal decomposition of organic molecules is very complicated and occurs in a few main stages. The first stage comprises physicochemical transformation (dehydration, melting, changes in conformation of molecules, initial defragmentation etc.) and occurs at low temperature. The processes of defragmentation and partial oxidation of the H atoms prevail mainly in temperature range to 400 °C. In the range above 500 °C, the peaks on DTG or DSC curves are due to processes thermo-oxidation of the H and N atoms and pyrolysis of charcoal.

**Fig. 8:**
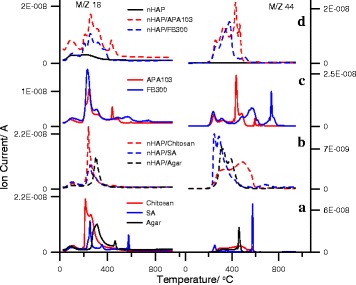
MS profile of H_2_O (18) and CO_2_ (44) versus temperature for initial PS and nHAp/PS composites

The low-temperature mass loss from 30 to 150 °C for PS and nHAp/PS corresponds to intact water desorption. The main weight loss was found for the PS degradation step (150–350 °C) [60, 61]. This step decomposition of organic molecules was confirmed by the increasing peaks for water (m/z 18) and carbon dioxide (m/z 44) in the mass spectra (Fig. 8) of analyzed samples. In nHAp/PS, condensation and elimination of hydroxyl groups occur at 150–250 °C [39]. TG and DTG curves of chitosan alone demonstrate the polymer chain decomposition from 197 to 276 °C with maxima at 211.8 and 237.6 °C. For composites nHAp/chitosan, only a single peak is observed with a maximum at 234.5 °C (Fig. 6a, Additional file 1: Table S1). This difference can be attributed to the changes in the structure or conformation of individual and adsorbed chitosan. TG and DTG curves of sodium alginate are characterized by decomposition of the polymer chain from 210 to 368.7 °C with maxima at 246.2 and 350.1 °C, which are most likely caused by condensation of hydroxyl groups and destruction of the organic component [62, 63]. For nHAp/sodium alginate, temperatures of peaks correspond to PS decomposition slightly shifted toward lower temperatures. This indicates some decrease in thermal stability of sodium alginate in the composite compared to sodium alginate alone. Decomposition of the polymer chain of agar occurs from 243 to 384 °C with a maximum at 297.1 °C. For nHAp/agar, the peak position corresponding to PS degradation does not practically change, but the width of the peak decreases.

TG and DTG curves of pectin FB300 characterize decomposition of the polymer chain in the first stage from 203 to 337 °C with maxima at 226.3 and 302.9 °C (Fig. 6c, Additional file 1: Table S1). For composites, three degradation peaks at 204.1, 250.1, and 316.2 °C are observed (Fig. 6d, Additional file 1: Table S1). The temperature range of PS degradation becomes wider compared with the pectin alone. The amount of physically adsorbed water is less in the composite than the pectin alone. Decomposition of pectin APA103 begins at lower temperatures than that of pectin FB300. The peak of pectin degradation in composites is slightly shifted toward lower temperatures.

Thermal effects upon degradation of polysaccharides can be estimated from the DSC data (Fig. 7). A weak endothermic peak between 50 and 150 °C with a maximum of ca. One hundred degree Celcius can be attributed to desorption of intact water. Thermodegradation of polysaccharides is usually accompanied by an exothermic effect. Typical DSC curves (Fig. 7) show three main peaks upon thermal analysis of sodium alginate and chitosan, and two peaks for agar. It is noteworthy that the intensive peaks distinguishable for initial (bulk) sodium alginate on DSC curve at 583.6 °C (Fig.7a) and DTG curve at 580.7 °C (Fig.6a) strongly changed for composite nHAp/SA: DSC peak disappears (Fig.7b), and DTG peak has much smaller intensity and shifted to temperature 672.7 °C (Fig.6b). The similar regularities are also observed for other composites HAp/PS: high-temperature peaks distinguishable on the DSC curves for bulk agar at 460.7 °C (Fig.7a), for bulk pectin FB300 at 746.7 and 586.6 °C, for bulk pectin APA103 at 593.3 °C (Fig.7c) are not observed on DSC curves for the corresponding composites nHAp/PS (Fig.7b, d). In the case of chitosan, all temperature peaks on DCS curve of bulk polysaccharide appear in DCS curve for the nHAp/chitosan composite but shifted to lower temperatures (Fig.7a, b). On DTG curves of composites agar/MS and pectin FB300/PS, the shift toward lower temperatures is observed for high-temperature peaks compared to the DTG curves for the initial polysaccharides: DTG peak for bulk agar at 457.5 °C shifted to 381.2 °C; peak on the DTG curve for pectin FB300 at 742.7 °C has disappeared on DTG curve for the composite; and the peak at 581.3 ° C shifted to 504.4 ° C. DTG peaks shifted slightly for nHAp/APA103 composites compared with initial pectin (Fig. 6c, d). Such peculiarities show that a strong interaction PS with nHAp results in a significant change in the thermal properties of PS. The multiple exothermic peaks of pectin degradation are observed at *T* > 150 °C, wherein for pectin FB300, it is not clear separation of peaks indicating the complexity and manifold of processes of degradation, while for pectin APA103 main peak is at 436 ° C.

### Adsorption of Sr(II)

Sr^2+^ ions occur in a non-hydrolized form in the aqueous solutions up to pH 10.5, since it does not form sparsely soluble oxides or hydroxides. Thus, Sr^2+^ ions are convenient to study the adsorption onto a surface of composites. The study of Sr^2+^ adsorption on HAp was described in detail previously [20]. The Sr^2+^ adsorption involves the surface hydroxyls of nHAp according to ion-change mechanism:

2 $$ 2\left(\equiv \mathrm{O}\mathrm{H}\right) + {\mathrm{Sr}}^{2+}\leftrightarrow {\left(\equiv \mathrm{O}\right)}_2\mathrm{S}\mathrm{r} + 2{\mathrm{H}}^{+} $$

**Fig. 9:**
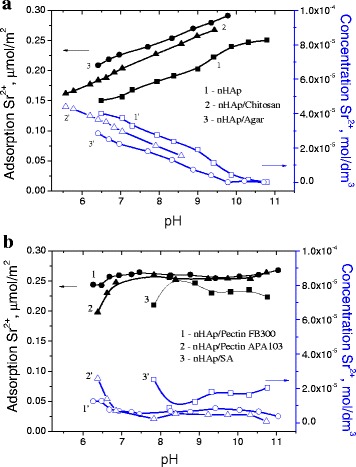
pH dependence of adsorption of ions Sr^2+^ from solution with concentration 0.0001 and 0.001 M NaCl (*black*) and the concentration Sr^2+^ remaining in the solution (*blue*) for **a** initial nHAp, nHAp/Chitosan and nHAp/Agar composites (4:1) and **b** nHAp/Pectin FB300, nHAp/Pectin APA103 and nHAp/SA composites (4:1)

The pH of solution is an important parameter that controls adsorption process because of ionization of surface functional groups and alteration of the solution composition. Figure 9 shows the pH dependences of the Sr^2+^ adsorption from 0.0001 M solutions for the initial nHAp and nHAp/PS composites. As it can be seen, a monotonic increase in the Sr^2+^ adsorption on initial nHAp is observed with increasing pH due to peculiarities of hydrolysis on the hydroxyapatite surface (Fig. 9a). The positively charged = CaOH_2_^+^ species and neutral = POH^0^ sites prevail in acidic solutions. Due to a high pH value, the surface of hydroxyapatite is deprotonated, releasing H^+^ ions in the solution and causing a shift of pH to lower value. The negatively charged = PO^−^ sites and neutral = CaOH^0^ sites predominate in alkaline solutions [64]. Adsorption of Sr^2+^ ions on the hydroxyapatite surface can proceed through the exchange of Ca^2+^ ions according to the reaction [20]:

3 $$ {\mathrm{Sr}}^{2+} + {\mathrm{Ca}}_5{\left({\mathrm{PO}}_4\right)}_3\mathrm{O}\mathrm{H}\ \leftrightarrow\ {\mathrm{Sr}\mathrm{Ca}}_4{\left({\mathrm{PO}}_4\right)}_3\mathrm{O}\mathrm{H} + {\mathrm{Ca}}^{2+} $$

The pH dependences of the Sr^2+^ adsorption vary for composites with different PS. For nHAp/agar and nHAp/chitosan, it is also observed a monotonic pH dependence of the Sr^2+^ adsorption, but the adsorption is higher than for the initial nHAp. The high values of the adsorption and a small remnant of the solution is achieved at a pH greater than 8 (Fig. 9a). For composites containing pectins, higher values of the adsorption are observed in the acidic pH range. In the case of nHAp/pectin FB300 composite, the Sr^2+^ adsorption reaches 0.25 μmol/m^2^ at pH 6.5 (Fig. 9b).

**Fig. 10:**
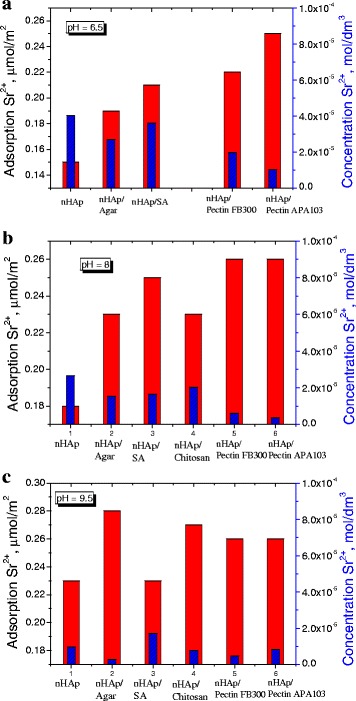
Adsorption of ions Sr^2+^ from solution with concentration 0.0001 and 0.001 M NaCl (*red*) and the concentration Sr^2+^ remaining in the solution (*blue*) for initial nHAp and all nHAp/PS composites (4:1) at different pH: **a** pH = 6.5, **b** pH = 8, and **c** pH = 9.5

Figure 10 shows a comparison of the adsorption values and a residue concentration of Sr^2+^ ions in the solution at pH 6.5, 8, and 9.5 for all composites. It can be seen that at pH 6.5, the composite containing pectin FB300 shows good Sr^2+^ adsorption, while at pH 8, the maximal adsorption values are inherent for nHAp/pectin FB300, nHAp/pectin APA103, and nHAp/SA. At pH 9, the composites containing chitosan and agar have the maximal adsorption.

For nHAp/PS, two mechanisms of the adsorption can be realized due to Sr(II) interactions with nHAp or polysaccharides. The adsorption of metal ions on polysaccharides occurs with participation of carboxyl groups of pectin, agar and sodium alginate, and amino groups in chitosan, which are capable of strong electrostatic interactions with metal ions [24–26, 35–37]. Therefore, all the composites studied show the adsorption capacity with respect to Sr(II) higher than the initial nHAp.

## Conclusions

Thus, nHAp/PS with different polysaccharides and different nHAp:PS ratios 4:1 and 1:1 synthesized by two-step process demonstrate certain decrease in the textural characteristics with increasing content of PS due to filling of inter-particle voids by polymers. However, the composites that have the HAP:PS ratio of 4:1 show relatively developed S_BET_ from 49 m^2^/g for nHAp/pectin FB300 to 82 m^2^/g for nHAp/SA. Mainly mesoporosity is the characteristic for the composites, since contribution of micro and macro pores is negligible. At the nHAp:PS ratio 1:1, a film-like structure was formed. The specific surface area and porosity largely depend on the nature of the polysaccharide, and maximal S_BET_ value of 43 m^2^/g is for nHAp/agar. The thermal properties of the composites show a certain influence of nHAp on polysaccharide degradation. However, composites have sufficient thermal stability. In the composites, the polysaccharide degradation occurs at temperatures above 200 °C. It was found out that for nHAp/PS composites at the component ratio 4:1, the adsorption capacity with respect to Sr^2+^ ions is higher than for the initial nHAp. This makes these composite promising for the use as adsorbents for metal cations from aqueous media.

## Additional file

Additional file 1: Table S1. Thermal behavior of studied samples (T_onset_, T_max_, T_end_ are an onset, maximum degradation, and end temperatures determined for DTG curves). (DOCX 20 kb)
