# Supplementary material for: Synthesis, Structural, and Adsorption Properties and Thermal Stability of Nanohydroxyapatite/Polysaccharide Composites
Source: Nanoscale Res Lett. 2017 Feb 27;12:155. doi: 10.1186/s11671-017-1911-5 (PMC5328890; doi:10.1186/s11671-017-1911-5)
Supplement: Additional file 1: Table S1. — Thermal behavior of studied samples (Tonset, Tmax, Tend are an onset, maximum degradation, and end temperatures determined for DTG curves). (DOCX 20 kb) [file 11671_2017_1911_MOESM1_ESM.docx]

**Additional file 1: Table S1** Thermal behavior of studied samples (T_onset_, T_max_, T_end_ are an onset, maximum degradation and end temperatures determined for DTG curves)

| **Sample** | **Thermal**  **Range,** °C | **Mass loss, %** | **T_onset_** | **T_end_** | **T_max_,**°C | **Assignment** |
| --- | --- | --- | --- | --- | --- | --- |
| Sodium Alginate | 30–150 | 7.78 |  |  | 73.0 | Water elimination |
|  | 150–350 | 38.93 | 224.4 | 262.5 | 246.2 | Organic decomposition |
|  |  |  | 335.6 | 368.7 | 350.1 |  |
|  | 350–950 | 26.22 | 568.4 | 590.6 | 580.7 | Burning of organics |
| nHAp/SA | 30–150 | 3.44 |  |  | 92.3 | Water elimination |
|  | 150–350 | 24.96 | 223.2 | 263.9 | 242.8 | Organic decomposition |
|  |  |  | 264 | 313.0 | 276.3 |  |
|  | 350–950 | 8.61 | 583 | 783 | 672.7 | Burning of organics |
| Agar | 30–150 | 4.6 |  |  | 85.2 | Water elimination |
|  | 150–350 | 52.3 | 243 | 384 | 297.1 | Organic decomposition |
|  | 350–950 | 33.42 | 419 | 488 | 457.5 | Burning of organics |
| nHAp/Agar | 30–150 | 1.6 |  |  | 87.0 | Water elimination |
|  | 150–350 | 22.8 | 271.8 | 358 | 297.1 | Organic decomposition |
|  | 350–950 | 8.64 | 358 | 449.9 | 381.2 | Burning of organics |
| Chіtosan | 30–150 | 4.6 |  |  | 99 | Water elimination |
|  | 150–350 | 51.82 | 197.0 | 220.3 | 211.8 | Organic decomposition |
|  |  |  |  | 275.8 | 237.6 |  |
|  | 350–950 | 34.56 | 400.9 | 525.0  561  583 | 468  550  568 | Burning of organics |
| nHAp/Chіtosan | 30–150 | 3.6 |  |  | 96.2 | Water elimination |
|  | 150–350 | 27.15 | 221.9 | 251.2 | 234.5 | Organic decomposition |
|  | 350–950 | 19.7 | 371.8 | 586.0 | 476.1 | Burning of organics |
| Pectin FB300 | 30–150 | 4.3 |  |  | 96.8 | Water elimination |
|  | 150–350 | 53.7 | 203.2 | 269.3 | 226.3 | Organic decomposition |
|  |  |  | 270.0 | 337.3 | 302.2 |  |
|  | 350–950 | 33.8 | 433.8  529.9 | 504.2  670.8 | 473.4  564.6 | Burning of organics |
|  |  |  | 716.8 | 777.1 | 737.8 |  |
| nHAp/ Pectin FB300 | 30–150 | 3.5 |  |  | 102.8 | Water elimination |
|  | 150–350 | 32.85 | 179.3 | 213.2 | 204.1 | Organic decomposition |
|  |  |  | 224.6 | 276.9 | 250.1 |  |
|  |  |  | 291.0 | 349.0 | 316.2 |  |
|  | 350–950 | 14.07 | 350 | 398.0 | 372.6 | Burning of organics |
|  |  |  | 471.2 | 549.0 | 506.0 |  |
| Pectin APA103 | 30–150 | 3.7 |  |  | 81.7 | Water elimination |
|  | 150–350 | 56.1 | 198.3 | 219.0 | 215.0 | Organic decomposition |
|  |  |  | 219.0 | 259.3 | 239.4 |  |
|  |  |  |  | 345.3 | 302.4 |  |
|  | 350–950 | 34.57 | 421.6 | 441.0 | 433.5 | Burning of organics |
|  |  |  | 441.0 | 508.1 | 474.2 |  |
|  |  |  | 582.4 | 606.5 | 594.5 |  |
| nHAp/ Pectin APA103 | 30–150 | 5.9 |  |  | 73.9 | Water elimination |
|  | 150–350 | 33.82 | 185.2 | 217.9 | 203.2 | Organic decomposition |
|  |  |  | 220.0 | 269.4 | 248.0 |  |
|  |  |  | 270.0 | 372.6 | 300.9 |  |
|  | 350–950 | 15.35 | 412.9 | 445.0 | 422.8 | Burning of organics |
|  |  |  | 451.3 | 473.8 | 460.8 |  |
